# A novel strategy for sibship determination in trio sibling model

**DOI:** 10.3325/cmj.2012.53.336

**Published:** 2012-08

**Authors:** James Chun-I Lee, Yen-Yang Lin, Li-Chin Tsai, Chun-Yen Lin, Tsun-Ying Huang, Pao-Ching Chu, Yu-Jen Yu, Adrian Linacre, Hsing-Mei Hsieh

**Affiliations:** 1 Department of Forensic Medicine, College of Medicine, National Taiwan University, Taipei, Taiwan ROC; 2 Institute of Forensic Medicine, Ministry of Justice, New Taipei City, Taiwan ROC; 3 Department of Forensic Science, Central Police University, Taoyuan, Taiwan ROC; 4 School of Biological Sciences, Flinders University, Adelaide, Australia

## Abstract

**Aim:**

To use a virtually simulated population, generated from published allele frequencies based on 15 short tandem repeats (STR), to evaluate the efficacy of trio sibship testing and sibling assignment for forensic purposes.

**Methods:**

Virtual populations were generated using 15 STR loci to create a large number of related and unrelated genotypes (10 000 trio combinations). Using these virtual populations, the probability of related and unrelated profiles can be compared to determine the chance of inclusions of being siblings if they are true siblings and the chance of inclusion if they are unrelated. Two specific relationships were tested – two reference siblings were compared to a third true sibling (3S trio, sibling trio) and two reference siblings were compared to an unrelated individual (2S1U trio, non-sibling trio).

**Results:**

When the likelihood ratio was greater than 1, 99.87% of siblings in the 3S trio population were considered as siblings (sensitivity); 99.88% of non-siblings in the 2S1U trio population were considered as non-siblings (specificity); 99.9% of both populations were identified correctly as siblings and non-siblings; and the accuracy of the test was 99.88%.

**Conclusions:**

The high sensitivity and specificity figures when using two known siblings compared to a putative sibling are significantly greater than when using only one known relative. The data also support the use of increasing number of loci allowing for greater confidence in genetic identification. The system established in this study could be used as the model for evaluating and simulating the cases with multiple relatives.

DNA profiling when applied to the identification of human remains requires comparison to reference data generated from either ante mortem samples or from known living relatives (1,2). If there are direct genetic relatives, such as an offspring and both genetic parents, then this process is well established; however if there are only putative siblings then the opportunity for misidentification increases. Such instances arose in the identification of deceased from a mass disaster (3). The comparison of the DNA profiles of two putative siblings can result in a likelihood ratio (LR) or combined sibling index (SI), being the odds favoring the tested samples originating from siblings compared to the samples originating from two genetically unrelated individuals. Previous studies have shown that the combined SI values will increase if more short tandem repeat (STR) loci are used (4,5) and reported a method for determining sibship (6). Recently, the universal algorithms for commonly used kinship indexes between two individuals have been established (7), and it has been suggested from the comparison study that the power of the identity by state (IBS) method was similar to that of ITO method (8) in full sibling determination, with advantages of convenience in calculation and independence on the allele frequency of STR loci (9). This study extends the data to cases where an unknown sample is tested and compared to two reference siblings.

Basic laws of inheritance are used to assess the probability that common alleles in the tested sample and a reference sample are inherited identical by decent (IBD). There is a 25% chance that two siblings will inherit both alleles IBD from common parents, a 50% chance that two siblings will inherit one parental allele IBD, and a 25% chance that two siblings will inherit IBD no parental alleles and therefore will share no alleles at a diploid locus. This last possibility, where there is a 25% chance that at any one locus the two true siblings may share neither allele, is problematic when identifying deceased in a mass disaster when there are only living siblings for genetic linkage. While the probability that one sibling will inherit 0 alleles IBD from their common parents is constant at 25% for each locus, the probability that two full siblings will not share an allele/locus partly depends on locus polymorphism information content (PIC) or heterozygosity. Greater PIC/locus will increase the chance that 2 siblings have neither allele in common (10). Additionally, greater confidence in sibling assignment of the tested sample might be obtained if there are 2 reference siblings for comparison. In such a situation, a sibling trio, the DNA type of the tested sample can be compared to those of the 2 reference siblings to determine the likelihood of either being a sibling or being genetically unrelated. This may provide confidence in an exclusion, based on sufficient inconsistencies, if there is no expected allele observed in the unknown (or tested) sibling. For inclusion, the LR will increase dramatically (10).

Previous studies (6-13) undertook DNA typing of known genetic relatives from which they made conclusions of putative sibling identification. These studies are necessarily limited by the availability of data. Computer-simulated populations have been also used (2,5,14) to generate virtual populations, with the limitation that these are generated rather than real data, but with the great benefit of an increase in the size of the data available. In this study, we report on the use of a virtual simulated population generated from published allele frequencies based on 15 STR to evaluate the efficacy of trio sibship testing and sibling assignment (15).

## Materials and methods

### Populations

Two simulated populations with genotypes of 15 STR loci were created from members of a previously described population (15) to produce trio sibling combinations. These data were generated by Microsoft^®^ Office Excel 2007. The two populations were designed to create two distinct situations: 2 reference siblings with a true sibling (3S trio, sibling trio) and 2 reference siblings with an unrelated individual (2S1U trio, non-sibling trio). Each population contained 10 000 trio data sets based on the 15 STR loci generated by the AmpFISTR^®^ Identifilier^TM^ kit (Applied Biosystems, Foster City, CA, USA). The population was created by starting with the assignment of alleles for both father and mother randomly chosen from the population data (15) in a spreadsheet of Microsoft^®^ Office Excel 2007. After genotypes of parents are assigned, genotypes of their offsprings can be assigned by randomly selecting one allele from each locus from each parent. Three offsprings from each “family” were selected to generate the 3S trio population. Two offsprings from each “family” were selected and combined with an unrelated individual to generate the 2S1U trio population.

### Calculations

The trio genotype combinations were entered into a spreadsheet and all calculations were performed using Microsoft^®^ Office Excel 2007. The likelihood ratio (LR) of each unique combination was calculated using related (R) and unrelated (U) hypotheses. R is the probability of observing the genotype of S3 given that reference siblings S1 and S2 are truly full siblings of S3. U is the probability of observing the genotype of S3 given that the reference genotypes of full siblings S1 and S2 are unrelated to S3. R is the sum of the genotype probabilities deduced from three siblings S1, S2, and S3, as shown in the example (Table 1). U is the sum of the possible probabilities of genotypes deduced from two siblings, S1 and S2, and the probability of an unrelated S3. All LR formulas of possible trio combinations were generated and built in the spreadsheet. The allele frequencies used are for the Taiwan population (15). As this was a virtual population, any substructure and possible mutational events were ignored. The distributions of likelihood ratio (LR) values were plotted using the SigmaPlot 9 (Systat software, Inc., San Jose, CA, USA), with the curve fitting analysis.

**Table 1:** The likelihood ratio formula of the example (2 reference siblings S1 and S2 and alleged sibling S3) generated by the method of family genotype comparison*

	Father		Mother		S1		S2		S3		Combined probability
	G	P	G	P	G	P	G	P	G	P	
R1	AB	2ab	AC	2ac	AA	1/4	AB	1/4	AC	1/4	2ab×2ac ×1/4×1/4×1/4
R2	AC	2ac	AB	2ab	AA	1/4	AB	1/4	AC	1/4	2ac×2ab ×1/4×1/4×1/4
U1	AB	2ab	AA	a^2^	AA	1/2	AB	1/2	AC	2ac	2ab×a^2^×1/2×1/2×2ac
U2	AA	a^2^	AB	2ab	AA	1/2	AB	1/2	AC	2ac	a^2^×2ab ×1/2×1/2×2ac
U3	AB	2ab	AX	2ax	AA	1/4	AB	1/4	AC	2ac	2ab×2ax×1/4×1/4×2ac
U4	AX	2ax	AB	2ab	AA	1/4	AB	1/4	AC	2ac	2ax×2ab×1/4×1/4×2ac
R/U = (R1+R2)/(U1+U2+U3+U4) = 1/(8a +8a^2^)											

*G and P designate genotype and probability. A, B, C, and X represent the alleles. a, b, c, and x represent the frequencies of allele A, B, C, and X respectively; the x value is 1-a. R1 and R2 represent the probabilities for two scenarios of the family genotypes deduced from S1, S2, and S3. U1 to U4 are the probabilities for four scenarios of the family genotypes deduced from S1, S2 and where S3 is unrelated.

Sensitivity is the proportion of tests indicating that S3 is a true sibling of S1 and S2 in the 3S population. Specificity is the proportion of tests indicating that S3 is unrelated to the combination of reference siblings S1 and S2 among 2S1U cases. A positive predictive value (PPV) was calculated as the true positive divided by the sum of true positives and false positives. The PPV indicated the proportion of samples identified correctly as siblings using the model of Gaytmenn et al (11). A negative predictive value (NPV) was calculated as the true negative divided by the sum of true negatives and false negatives. The NPV indicated the proportion of subjects identified correctly as non-siblings (11). Accuracy was determined as the true positive divided by the total values (sum of the true positive, false positive, and false negative).

## Results

Initial results generated 64 genotype combinations resulting from 2 reference siblings and one tested sample. Within these combinations, there were 37 combinations with common parents where S3 could not be excluded as being a sibling of the other 2 reference siblings (S1 and S2) in trio sibship test (Table 2). The LR formulas for these combinations (Table 3) were generated according to standard methods of genetic inheritance (Table 1). For the other 27 combinations, sample S3 within the trio did not possess the expected alleles and fell into the category of an exclusion (or genetic inconsistency) as being a sibling of the other two (Table 4).

**Table 2:** Genotype combinations in the 3S population in which the alleged sibling (S3) cannot be excluded as being a sibling of the other 2 reference siblings (S1 and S2) in trio sibship test*

S1	S2	S3	S1	S2	S3	S1	S2	S3
AA	AA	AA	AA	BC	AA	AB	AC	AA
AA	AA	AX	AA	BC	AB	AB	AC	AB
AA	AA	XX	AA	BC	AC	AB	AC	AC
AA	AA	XY	AA	BC	BC	AB	AC	BB
AA	AB	AA	AB	AB	AA	AB	AC	BC
AA	AB	BB	AB	AB	AB	AB	AC	CC
AA	AB	AB	AB	AB	BB	AB	AC	CX
AA	AB	AX	AB	AB	AX	AB	AC	BX
AA	AB	BX	AB	AB	BX	AB	CD	AB
AA	BB	AA	AB	AB	XX	AB	CD	AC
AA	BB	BB	AB	AB	XY	AB	CD	AD
AA	BB	AB				AB	CD	BC
						AB	CD	BD
						AB	CD	CD

*Alleles X and Y in S3 are those not observed in siblings S1 and S2.

**Table 3:** The formulas to derive the likelihood ration (LR) for 37 non-excluded combinations in a trio sibling test with 2 reference siblings (S1 and S2) and one putative sibling (S3)

S1	S2	S3	LR formula
AA	AA	AA	(1 + 6a +9a2)/(4a2 + 8a3 + 4a4)
AA	AA	AB	(1 + 3a)/(4a +8a2 + 4a3)
AA	AA	BB	1/(4 + 8a +4a2)
AA	AA	BC	1/(4 + 8a +4a2)
AA	AB	AA	(1 + 3a)/(4a2 + 4a3)
AA	AB	BB	1/(4b +4ab)
AA	AB	AB	(1 + 3a)/(8ab +8a2b)
AA	AB	AC	1/(8a +8a2)
AA	AB	BC	1/(8b +8ab)
AA	BB	AA	1/(4a2)
AA	BB	BB	1/(4b2)
AA	BB	AB	1/(4ab)
AA	BC	AA	1/(4a2)
AA	BC	AB	1/(8ab)
AA	BC	AC	1/(8ac)
AA	BC	BC	1/(8bc)
AB	AB	AA	(1 + 3a)/(4a +4ab +4a2 + 4a2b)
AB	AB	AB	(1 + 3a +3b +9ab)/(8ab +8a2b +8ab2 + 8a2b2)
AB	AB	BB	(1 + 3b)/(4b +4ab +4b2 + 4ab2)
AB	AB	AC	(1 + 4a)/(8a +8a2 + 8ab +8a2b)
AB	AB	BC	(1 + 4b)/(8b +8ab +8b2 + 8ab2)
AB	AB	CC	1/(4 + 4b +4a +4ab)
AB	AB	CD	1/(4 + 4a +4b +8ab)
AB	AC	AA	1/(4a +8a2)
AB	AC	AB	(1 + 4a)/(8ab +16a2b)
AB	AC	AC	(1 + 4a)/(8ac +16a2c)
AB	AC	BB	1/(4b +8ab)
AB	AC	BC	(a + b)/(8bc +16abc)
AB	AC	CC	1/(4c +8ac)
AB	AC	CD	1/(8c +8ac)
AB	AC	BD	1/(8b +8ab)
AB	CD	AB	1/(8ab)
AB	CD	AC	1/(16ac)
AB	CD	AD	1/(16ad)
AB	CD	BC	1/(16bc)
S1	S2	S3	LR formula
AB	CD	BD	1/(16bd)
AB	CD	CD	1/(8cd)

*a, b, c, and d represent the frequencies of allele A, B, C, and D respectively.

**Table 4:** Genotype combinations in which the alleged sibling (S3) can be excluded as being a sibling of the other 2 reference siblings (S1 and S2) in trio sibship test*

S1	S2	S3	S1	S2	S3	S1	S2	S3
AA	AB	XX	AA	BC	CC	AB	CD	BB
AA	AB	XY	AA	BC	CX	AB	CD	BC
AA	BB	AX	AA	BC	XX	AB	CD	BX
AA	BB	BX	AA	BC	XY	AB	CD	CC
AA	BB	XX	AB	AC	AX	AB	CD	CX
AA	BB	XY	AB	AC	XX	AB	CD	DD
AA	BC	AX	AB	AC	XY	AB	CD	DX
AA	BC	BB	AB	CD	AA	AB	CD	XX
AA	BC	BX	AB	CD	AX	AB	CD	XY

*Alleles X and Y in S3 are those not observed in siblings S1 and S2.

**Figure 1:**
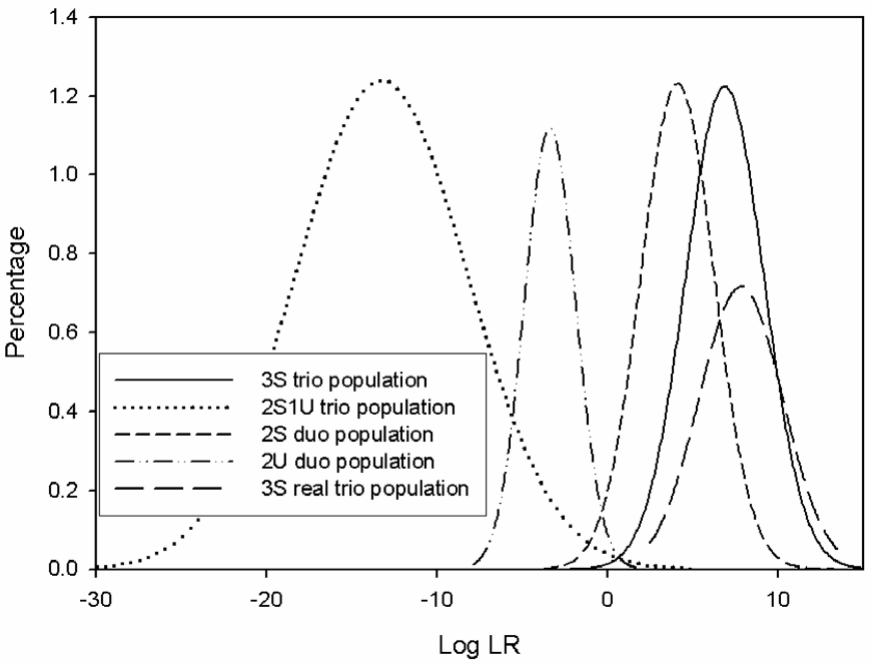
The distribution of likelihood ratio (LR) values is shown for the 3S real trio population, and 2U, 2S, 3S, and 2S1U simulated populations. The x-axis represents the log10 of the LR and the y-axis represents the percentage of combinations. The mean ± standard deviation of the above populations were 7.8524 ± 2.453, -3.3624 ± 1.5062, 4.1235 ± 2.1517, -13.2596 ± 5.0295, and 6.918 ± 2.2997, respectively.

The LR values and distribution for populations 3S and 2S1U were calculated (Figure 1). For this calculation, the 27 trio combinations (Table 4), where there was a genetic inconsistency, were given a value of 0.001, rather than zero. The log LR values of population 3S ranged from -2.24 to 15.96 and for population 2S1U they ranged from -32.57 to 2.15. The simulated method was validated by using 75 real trio siblings, and the log LR ranged from 2.33 to 13.20. This distribution for the real population showed concordance with the simulated population 3S (Figure 1), indicating the accuracy of the simulation model. In Figure 1, the 2U and 2S duo populations (one known sibling compared to one tested sample) were also included and used as the simulated controls. The distributions of log LR were closer for 2U and 2S than for 2S1U and 3S. These data indicated that greater confidence in sibling assignment of the tested sample could be obtained if there were two reference siblings rather than one for comparison.

**Figure 2:**
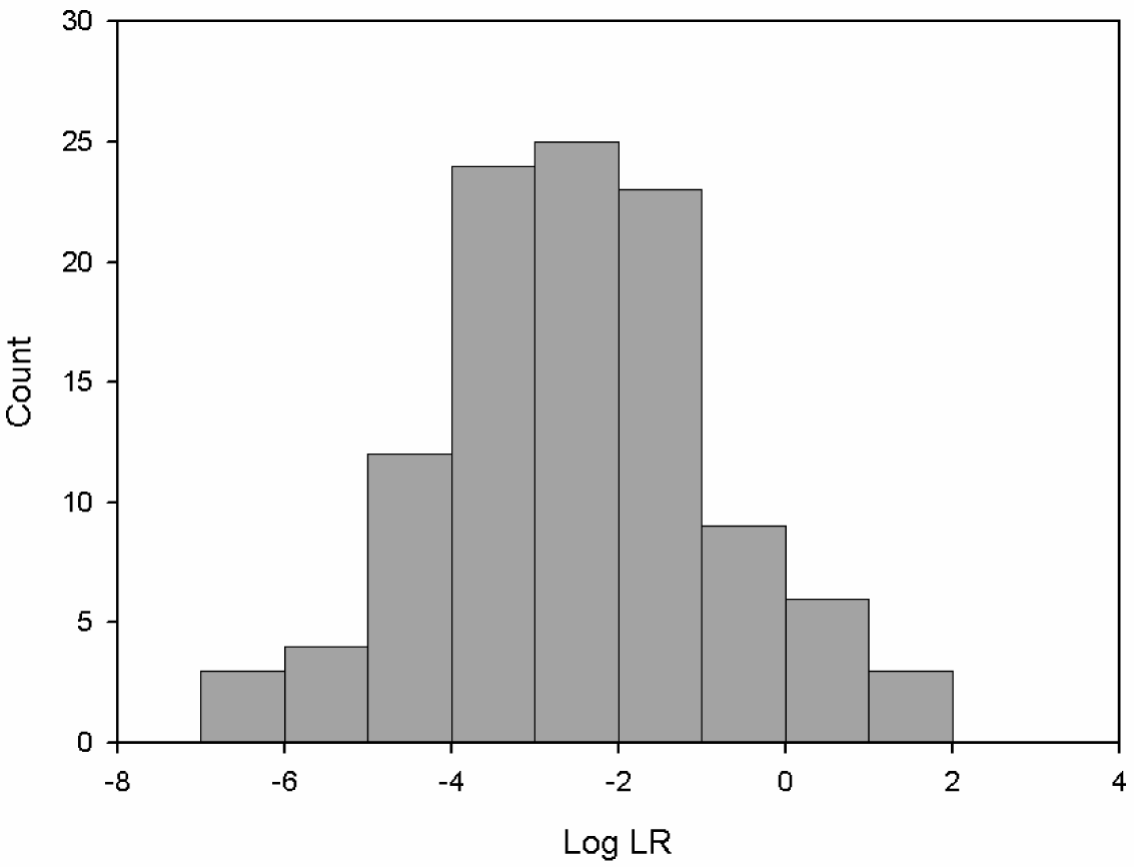
The distribution of likelihood ratio (LR) values is shown for the 109 combinations in the 2S1U population that were not excluded as being possible trios.

The data for the 2S1U trio population showed that there was only 1.09% (109 in 10 000) of the genotype combinations where the unrelated individual in the trio was not excluded as being a sibling. These data indicated that a test for sibship based on these genotype combinations would result in 98.91% of non-sibling trios being excluded correctly. The distribution of LR (log) values for these combinations that were not excluded in the 2S1U population ranged from -6.49 to 1.83 (Figure 2). Analysis of the number of loci indicating sibship exclusion showed that out of the 10 000 combinations there were 520 instances of the 2S1U scenario, with only one locus exhibiting a genetic inconsistency. These data highlight a possible risk of making a positive sibship identification based on one genetic inconsistency, such as an assumption of this being the result of a mutation. There were 1432 and 2111 instances for two and three loci exhibiting a genetic inconsistency, respectively. The STR loci of D18S51, D2S1338, and FGA were found to be most informative in excluding sibship. The power of exclusion (PE) (16) for nonparent of these loci was 0.727, 0.726, and 0.722 respectively (15). The PE of the least informative locus TPOX was only 0.338.

The sensitivity and specificity of the test was determined by comparing the populations 3S and 2S1U generated using these 15 STR loci. Table 5 shows the results for the trio siblings, where an LR of at least 100 was obtained; we used 100 as this was a figure suggested by the AABB for non-exclusions (17). The sensitivity, specificity, and accuracy were 98.50%, 99.99% and 99.25%, respectively; with a PPV of 99.99% and NPV of 98.52%. If an LR threshold value was greater than 1, 99.87% of siblings in the 3S trio population were considered as siblings (sensitivity); 99.88% of non-siblings in 2S1U trio population were considered as non-siblings (specificity); 99.9% of both populations were identified correctly as siblings and non-siblings; and the accuracy of the test was 99.88%. If the LR value was greater than 1000, then there were no expected false inclusions as the specificity was 100%, with an accuracy of 97.83%.

**Table 5:** The value of test result in trio siblings with the likelihood ratio (LR) of at least 100

Test result	True status		Total
	sibling	unrelated	
Sibling	9850	1	9851
Unrelated	150	9999	10 149
Total	10 000	10 000	20 000

### Application to a real-world case

An illustration of the application of a combined SI is provided (Table 6) in the following real case where a putative third sibling (S3) was tested against two confirmed siblings (S1 and S2). The figure obtained for the combined SI using the trio sibling test (36 561 850.243) was far higher than for the duo test (4163.289 or 3977.735).

**Table 6:** Case study of the combined sibling index (SI) in duo and trio sibling situations from a family of 2 true siblings (S1 and S2) and a putative third sibling (S3)

STR loci	S1	S2	S3	SI between S1 and S3	SI between S2 and S3	SI between S1, S2, and S3
D8S1179	12,17	13,15	12,15	1.22	1.002	2.917
D21S11	29,31	29,30.2	30.2,31	1.457	12.876	8.381
D7S820	8,11	8,11	8,11	4.353	4.353	5.265
CSF1PO	12,13	9,11	9,11	0.25	15.228	11.631
D3S1358	15,17	15,15	17,17	1.315	0.25	0.791
TH01	9,9	9,9	9,9	2.412	2.412	2.997
D13S317	10,11	10,12	10,11	5.233	1.113	4.406
D16S539	12,12	11,12	9,12	1.423	0.836	0.483
D2S1338	20,20	22,25	20,25	2.492	2.194	17.435
D19S433	13,15	13,13	13,15	8.483	1.075	9.11
VWA	14,17	17,18	14,17	3.403	0.757	2.824
TPOX	11,11	8,11	8,11	1.121	1.697	1.474
D18S51	13,15	15,19	13,15	5.426	0.929	4.82
D5S818	7,12	11,12	11,12	0.833	3.071	2.396
FGA	21,21	21,22	19,22	0.25	0.959	0.634
AMEL	X,Y	XX	XX			
Combined SI				4163.289	3977.735	36 561 850.243

## Discussion

We studied sibship assignment based on 15 STR loci; where the tested sample was a putative sibling of 2 reference siblings. The use of a virtual population starting with known alleles allows for up to 10 000 trio combinations to be used in such a study. In addition to the high exclusion rate for non-siblings, this study showed a high degree of sensitivity, specificity, and accuracy in sibling identification. In our previous study (14), the variations of the distribution for paternity index (PI) and random man not excluded (RMNE) values in paternity test were evaluated based on 1244 virtual families. There were minor variations between the PI and 1/RMNE values in trio parentage testing compared with duo parentage testing. Also, the distribution of PI/(1/RMNE) for duo families exhibited greater variation than that for trio families. This highlighted the effect that different mathematical methods can have on the results using either of these tests; this effect was found to be greater in the duo cases. A consequence is that with more individuals being tested greater confidence in the results will be obtained.

Our data for trio situations are much higher than those reported previously in duo sibship tests (11,12,18). In the report using 33 duo pairs and 15 STR loci, sensitivity of 93.94% and specificity of 90.91% was reported (19). This study provides evidence that analysis of trio sibship testing with a fixed number of STR loci is more powerful than analysis of duos using the same loci. The trio sibling model described is a cost-effective way to screen disaster samples for sibling assignment and identification. The system established in this study could be used as the model for evaluating and simulating the cases with multiple relatives.
